# Supplementary material for: Targeting of Silver Cations, Silver-Cystine Complexes, Ag Nanoclusters, and Nanoparticles towards SARS-CoV-2 RNA and Recombinant Virion Proteins
Source: Viruses. 2022 Apr 26;14(5):902. doi: 10.3390/v14050902 (PMC9144282; doi:10.3390/v14050902)
Supplement: Supplementary file 1 [file viruses-14-00902-s001.zip › viruses-1677170-supplementary.pdf]

Supplementary Data

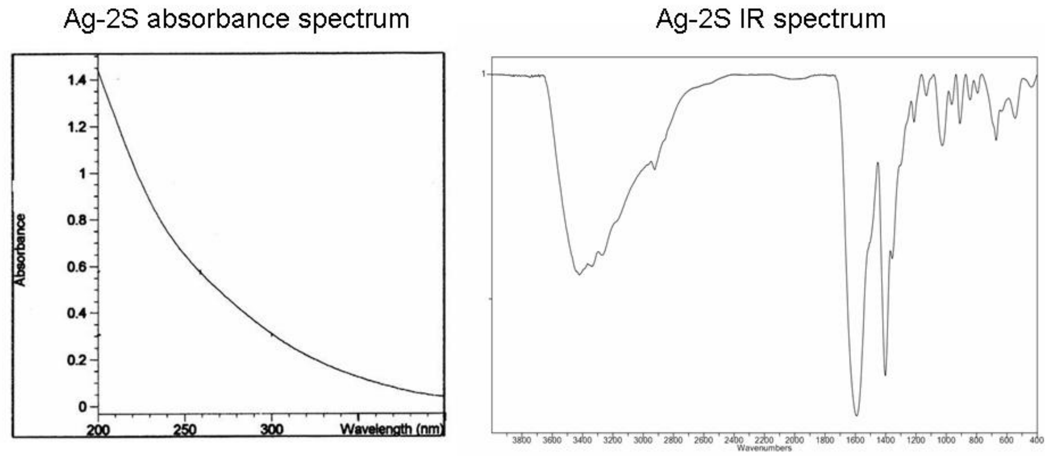

Figure S1. Physico-chemical properties of Ag-2S complex: UV absorbance and IR-spectrum.

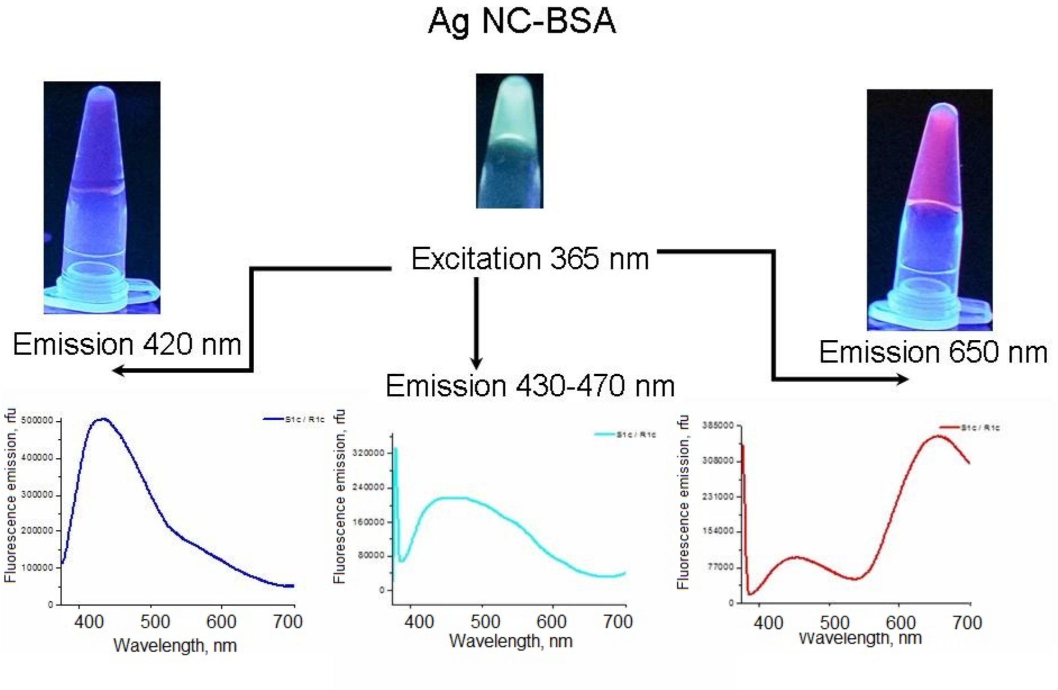

Figure S2. Fluorescence emission spectra of Ag NC with BSA.

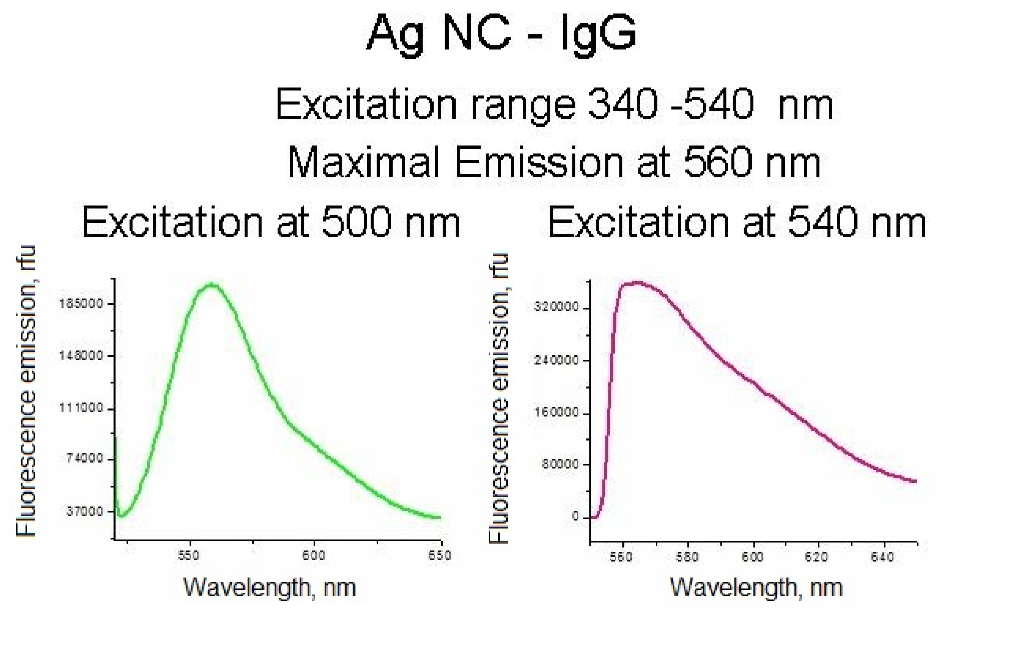

Figure S3. Fluorescence emission spectra of Ag NC with IgG.
